# Encoding of everyday objects in older adults: Episodic memory assessment in virtual reality

**DOI:** 10.3389/fnagi.2023.1100057

**Published:** 2023-03-13

**Authors:** Marlon O. Pflueger, Ralph Mager, Marc Graf, Rolf-Dieter Stieglitz

**Affiliations:** ^1^University of Basel, Forensic Clinic of the University Psychiatric Clinics, Basel, Switzerland; ^2^University of Basel, University Psychiatric Clinics, Basel, Switzerland

**Keywords:** virtual-reality, ecological validity, episodic object memory, aging, serial/semantic clustering, memory association network, small-world network

## Abstract

**Introduction:**

Age-related decline in episodic memory performance in otherwise healthy older adults is indisputably evident. Yet, it has been shown that under certain conditions episodic memory performance in healthy older adults’ barely deviates from those seen in young adults. Here we report on the quality of object encoding in an ecologically valid, virtual-reality based memory assessment in a sample of healthy older and younger adults with comparable memory performance.

**Methods:**

We analyzed encoding by establishing both a serial and semantic clustering index and an object memory association network.

**Results:**

As expected, semantic clustering was superior in older adults without need for additional allocation of executive resources whereas young adults tended more to rely on serial strategies. The association networks suggested a plethora of obvious but also less obvious memory organization principles, some of which indicated converging approaches between the groups as suggested by a subgraph analysis and some of which indicated diverging approaches as suggested by the respective network interconnectivity. A higher interconnectivity was observed in the older adults’ association networks.

**Discussion:**

We interpreted this as a consequence of superior semantic memory organization (extent to which effective semantic strategies diverged within the group). In conclusion, these results might indicate a diminished need for compensatory cognitive effort in healthy older adults when encoding and recalling everyday objects under ecologically valid conditions. Due to an enhanced and multimodal encoding model, superior crystallized abilities might be sufficient to counteract an age-related decline in various other and specific cognitive domains. This approach might potentially elucidate age-related changes in memory performance in both healthy and pathological aging.

## 1. Introduction

Due to considerable progress in imaging technologies, the aims and purposes of clinical neuropsychology have changed a great deal. Lesion localization, once a major professional objective of neuropsychologists, has now become negligible (Chaytor and Schmitter-Edgecombe, 2003; Spooner and Pachana, 2006). However, neuropsychology has not yet lost its clinical significance. Instead, in addressing concerns such as everyday functionality, neuropsychology continues to play a major and unique role in the comprehensive assessment of patients suffering from various brain diseases and head injuries. Consequently, the administration and development of ecologically sound diagnostic tools and test procedures are mandatory. The ever faster advancing virtual reality (VR) and gaming technologies might facilitate the development of such tools and procedures. In fact, by recognizing the 125th anniversary of the American Psychological Association, a special issue of the journal Neuropsychology emphasizes that these technologies are beginning to have an impact on the field (Brown et al., 2017). VR environments integrate the rigor of laboratory experimental control with multi-modal and context-embedded tasks that more closely resemble everyday-related cognitive demands (Parsons, 2015). Therefore, VR technologies have the potential to easily develop new neuropsychological assessments with substantial ecological validity in terms of a verisimilitude approach (Spooner and Pachana, 2006).

Currently, numerous studies deploying VR environments have already been reported, addressing various cognitive subsystems (e.g., Broeren et al., 2007; Banville et al., 2010; Bioulac et al., 2012; Plancher et al., 2012; Díaz-Orueta et al., 2014) in varying scenarios (e.g., Rizzo et al., 2000, 2009; Matheis et al., 2007; Rand et al., 2007) and demonstrating both considerable heterogeneity and sensitivity in discriminating intact from impaired cognition. Moderator analyses indicated age, the type of clinical population, the degree of interactivity, and the presence of distracters as the potential source of heterogeneity but not gender, performance indicators, or type of the VR platform (Neguț et al., 2016). However, besides a great deal of heterogeneity, evidence from VR studies occasionally indicates counter-intuitive results. More precisely, some VR-related findings may apparently contradict already established results, which were replicated repeatedly. Matheis and colleagues, e.g., reported a comparable rate of learning and recall performance in healthy controls and a subgroup of patients suffering from a traumatic brain injury when assessed by an ecologically valid VR environment even though performance differences in the traditional assessments of episodic memory remained evident (Matheis et al., 2007). Similarly, we reported on the performance of healthy older adults compared with the performance of younger adults on episodic memory tests conducted in a real-life resembling VR environment (Pflueger et al., 2018). Thus, the observed heterogeneity of VR study results might not only be due to moderator variables *per se* but rather might also emerge as an inherent consequence of ecologically sound assessments. It is exactly this issue that deserves more clarity as pointed out by a recent review of VR in episodic memory research (Smith, 2019). Smith (2019), among others, concludes: of particular interest is the in-depth exploration of encoding in VR as opposed to analogous real-life settings.

We are reporting here on the quality of object encoding in a VR-based episodic memory assessment using a kitchen scenery in a sample of healthy older adults (HOAs) and younger adults (YAs). As previously reported by Pflueger et al. (2018), age-related memory deficits did not appear that evident during the VR-based assessment as expected by the performance in the traditional tests of episodic memory. Currently, we are recurring on the same set of data in order to perform an in-depth analysis of how encoding occurred in both the HOA and the YA. According to our previously drawn conclusions, we hypothesize that HOA makes more intense use of semantic strategies than YA does. Reversely, YA are relying more on the superior rote memory and, thus, produces more serial clustering or extended serial position effects than HOA. We do not assume the coding strategies to be completely different between the groups. Therefore, we are expecting a considerable overlap in the use of semantic strategies. We are going to show that exploratively by adopting an association graph approach.

## 2. Materials and methods

### 2.1. Participants and VR procedure

Inclusion criteria were subjectively sensed health and age-appropriate cognition. Exclusion criteria extended to below normative cognitive test performance; a history of neurological, psychiatric, toxic, metabolic, or systemic disease; general anesthesia; or the loss of consciousness > 15 min within the previous 6 months from the time of examination. Study participation required visiting the lab two times. During the first visit, the respective prerequisites for study participation were verified, and a comprehensive neuropsychological assessment was conducted. During the second visit, the VR memory examination was administered. The study was conducted in accordance with the Declaration of Helsinki (World Medical Association, 2001) and approved by the local ethics committee of the University of Basel, Switzerland, and all participants (18+) gave written informed consent.

The VR system was composed of a 4 × 2.3 m stereoscopic full HD projection system. The stereoscopic (3D) projection and the head-tracking facility required the participant to wear active shutter glasses, to which positional tracking markers were attached. The head-tracking system was used to localize the participant’s spatial head position and track changes in head orientation to adapt accordingly to the perspective of the screen projection. The VR itself was designed as a kitchen with multiple compartments concealing various objects. In total, 46 objects were used and each object occupied a unique position. The objects belonged either to a set of prototypical kitchen items (e.g., pan, fork, and bench scraper) or to non-kitchen items (e.g., keyboard, stethoscope, and pillow). Subsequent to an initial orientation phase in which participants had the opportunity to get acquainted and acquire a spatial representation of the kitchen by freely inspecting it, the memory examination was conducted.

The memory examination consisted of a three trial learning phase, where a total of 46 objects were consecutively presented by opening the respective compartment and by naming it by voice-over. Across all learning trials, the full set of objects was presented in a fixed and pseudo-randomized order, thus, following a previously determined presentation list. After completing the object presentation, the participants were asked to recall as many objects as they could remember in any order. The VR memory examination was completed by a short and long delay-free recall (approximately 25 min later), a spatial location cued-recall, and, finally, a recognition task. Study supervisors were in charge of the protocol of the participants’ responses across learning and recall trials in the exact order they were given. The remembered objects were then recoded and substituted by a number indicating their serial position in presentation order. For more detailed information regarding the recruitment procedure, the neuropsychological test battery was used and also VR memory examination including a comprehensive description of the participants, see Pflueger et al. (2018) and Supplementary material (Section 1 describes the sample description, Section 2 shows an example of a memory protocol from a single learning trial, and Section 2.2.1 shows a complete list of the objects presented).

As to decide whether the hypotheses held true or not, we generated both a semantic and serial clustering index. By means of these indices, a quantification of semantically and serially driven encoding strategies is possible. In addition, we constructed group-related association graphs of the most important object associations based on the recorded protocols during the VR memory examination. By means of these graphs, we were able to determine additional converging as well as diverging structural- and content-related properties of object encoding across the respective age groups. With content-related properties, we refer to object associations, which might be similar or different between the groups. With structural properties, we address network topological features, such as interconnectivity.

### 2.2. Determining the serial and semantic clustering index

According to the second and revised edition of the California Verbal Learning Test (CVLT), we made use of list-based clustering indices (Delis, 2000). List-based clustering (LBC) indices are basically defined as differences between observed LBC (LBC_OBS_) vs. expected LBC (LBC_EXP_) by chance (Stricker et al., 2002). The LBC_OBS_ is incremented, if serially or semantically adjacent objects/memory items are recalled consecutively. Whereas the first is obvious (recall of objects from serially adjacent list positions), the latter deserves some additional consideration. Memory items from the widely known CVLT are derived from predefined semantic categories. Thus, the decision of whether any two consecutively recalled items are semantically adjacent can easily be made. However, the objects from the VR memory examination were not selected according to predefined semantic categories. Therefore, a reference describing semantic relationships between these objects had to be established *post-hoc*. For this purpose, we used the GermaNet (see Supplementary material), which is a lexical-semantic database that relates German nouns, verbs, and adjectives semantically by grouping lexical units that express the same concept and by defining semantic relations between them (Hampel and Feldweg, 1997; Henrich and Hinrichs, 2010). By means of the GermaNet, we were able to derive a semantic similarity measure (Lin, 1998) for each pairwise combination (1035) of the entire object collection used. As to determine the semantic reference, in turn, we created an undirected graph where all the objects (vertices) were completely connected by weighted edges reflecting the derived semantic similarity. Finally, the semantic reference was compiled by traversing the graph such that the sum of the semantic similarity on the traversed path is globally maximized. This approach requires the solution of the so-called traveling salesman problem (refer to Supplementary material). With the semantic reference list at hand, the definition of what applies to the semantic LBC_OBS_ was straightforward: every consecutive recall of any two objects also adjacently found in the reference list incremented the semantic LBC_OBS_ by a value of one.

The LBC_EXP_ for both the semantic and the serial indices resulted from combinatorial and probabilistic principles and equated to LBC_EXP_ = 2*(*r*–1)/*N*, where r is the number of correctly recalled objects and N is the total number of objects presented in the VR memory examination. Thus, the maximal value of LBC_EXP_ that can be achieved amounts to 2*45/46 = 1.96 (see Supplementary material).

Once the clustering index is computed (LBC_OBS_–LBC_EXP_), its interpretation is straightforward. A positive index indicates the amount of semantic or serial clustering above chance, which maximally amounts to 45–1.96 = 43.04. For detailed information, refer to Supplementary material.

### 2.3. The generation of association graphs from memory protocols

As to determine the degree of similarity/dissimilarity of the encoding strategies used, association graphs were generated for both the HOA and the YA. The association graphs were established on memory protocols coding for the serial positions of successfully recalled objects across all learning and delayed recall trials. Thus, the vertices of the resulting graphs represented objects/memory items and the edges connecting these indicated association strength (see Supplementary material for a more detailed description of graph properties). By visualizing a network of the most important group-specific object associations, encoding properties might emerge that are not easily quantified or even determined otherwise. Moreover, structural properties of the network might reflect memory organization principles capable of bolstering recall performance (e.g., small-world property).

In order to create a respective association graph, the equality of an arbitrary graph representation with its adjacency matrix was exploited, i.e., a matrix whose elements determine whether a pair of vertices in the graph are neighbors (have an edge) or not. Rows and columns of the adjacency matrix reflect the vertices (objects) of the association graph and its elements encode absolute and pairwise recall frequencies of the respective vertices (objects; see Supplementary material). In order to render these frequencies comparable between groups of different sample sizes and to allow for an interpretation with respect to random chance, we determined the expected frequencies for each element of the matrix according to principles of the simple chi-squared test statistics, which assumes none-contingency between the vertices. In computing expected vs. observed frequency ratios, we obtained association likelihoods where a small value indicated a low probability of association by chance (see Supplementary material for a more detailed description of graph construction).

The resulting graph might be densely packed with multiple edges. The probability that every vertex is a neighbor of all other vertices in a graph increases fast with increasing sample size. This adds a lot of white noise to the associations of interest. Thus, we pruned the graph such that only the most important edges remained. Pruning reduces the number of edges such that, beginning with the least important ones, increasingly more important edges are removed as long as the graph remains connected. Incidentally, this procedure optimizes the minus log-likelihood of the graphs.

As to determine converging encoding strategies between the groups, we computed an intersection graph from both the HOA and the YA association graphs. The resulting cliques indicated common subgraphs or components of object associations. These cliques, then, served for coloring the vertices. The cliques were analyzed according to memory organization principles such as similarity of object location, context specificity (novelty), and other qualitative properties.

Finally, the association graphs were exploratively analyzed in terms of potential group differences between structural components such as edge weights (associative likelihood), serial position effects, serial clustering, semantic similarity, and complexity (i.e., mean vertex degree, local and global clustering of vertices, and path length). Moreover, a small-world index σ was calculated so as to compare the interconnectivity between the graphs (Watts and Strogatz, 1998; see Supplementary material). A graph can be said to belong to a class of small-world networks if it holds that *σ* > 1 (Humphries and Gurney, 2008). The small-world property indicates a highly efficient network in terms of its system dynamics, i.e., the efficiency with which different nodes in different parts of the graph might be accessed and retrieved. It is not an all-or-none property and it might be differentially graded across different networks. Moreover, it has been shown that small-worldness and the number of edges scale log-linearly with a vertex count. Thus, a network of a given size might be qualified by more or less expected small-worldness (see Supplementary material).

In order to demonstrate the validity of the association graph approach, we conducted an analysis of the associations between the edge weights (association likelihoods) and Lin’s semantic similarity measure (see Supplementary material).

### 2.4. Statistical analysis

Statistical analyses were accomplished using the R statistical computing environment (R Core Team, 2019). To compare descriptive data at a group or graph level, simple non-parametric Wilcoxon and chi-square tests were performed. In order to model both the semantic and the serial clustering indices, a mixed-effect model approach was conducted. The intercept and, where necessary, the slope were modeled as random factors. In considering the fixed effects, a sequential variable introduction and step-down approach were adopted. In addition to group and demographic variables, intelligence and other neuropsychological variables, especially the general executive capacity (Pflueger et al., 2018), were considered covariates. Variance inflation was monitored and kept below a value of 1.5. Both the Akaike and Bayesian information criteria were used to resolve which two competing models were superior. As to qualify for “goodness of fit,” an R2 measure was provided (Nakagawa and Schielzeth, 2013). The association graphs were created with an R package from (Gentleman et al., 2018) and analyzed with algorithms from (Hoos and Stützle, 2014; Carey et al., 2018), and (Hahsler and Hornik, 2007). By means of regression analysis, the edge weights were validated and compared between HOA and YA.

## 3. Results

### 3.1. The serial and semantic clustering indices

#### 3.1.1. The serial clustering index

In general, serial clustering was not used extensively by the participants. In the HOA index, values ranged between −0.2 in learning trial one and 0.87 in learning trial two, and in YA between 0.17 as in learning trial one and 0.89 in learning trial two and short delay free recall (sdfr), respectively. Thus, median difference effect sizes across all learning and recall trials were consequently small to very small, as shown in Table 1. The modeling approach resulted in a simple mixed model comprising a random intercept and one fixed effect (conditional *R*2 = 0.531). The fixed effect extended to an interaction of groups with a trial, where the trial was centered and modeled as a fourth-degree polynomial (*F*2, 207.3 = 6.0; *p* = 0.003). There was no effect of demographic variables, general executive capacity, or intelligence. The variable trial not only encompassed the learning trials from the VR memory examination but also the short and long delay-free recall, thus, ranging numerically from one to five. The dependent variable serial clustering index was log-transformed. The interaction of group and trial indicated a somewhat lesser use of serial strategies in HOA (t 214.2 = −3.1, *p* = 0.002) as compared with YA (*t* 204.7 = −1.8, *p* = 0.080) across learning and recall trials by less than half of a clustering unit. However, even though statistically significant, it should be noted that the effect is not very substantial (marginal *R*2 = 0.026).

**Table 1:** Median and interquartile range of the serial clustering index.

	HOA		YA		Effect size^a^	p^b^
	*N* = 18		*N* = 30			
	Median	IQR	Median	IQR		
trial1	−0.20	[−0.61;1.12]	0.17	[−0.55;1.47]	−0.13	0.354
trial2	0.87	[0.03;1.88]	0.89	[−0.25;2.08]	−0.04	0.782
trial3	0.65	[−0.16;1.77]	0.59	[−0.16;2.49]	−0.04	0.798
sdfr	0.83	[−0.04;1.55]	0.89	[−0.09;2.00]	−0.04	0.724
Ldfr	−0.17	[−0.62;0.76]	0.63	[0.07;2.17]	−0.23	0.110

a An effect size derived from the Wilcoxon z [z/sqrt(n)].

b Wilcoxon rank sum test.

HOA, healthy older adults; YA, young adults; IQR, interquartile range.

#### 3.1.2. The semantic clustering index

As a prerequisite, the calculation of the semantic clustering index requires a semantically optimized object reference list. This reference list could have been easily obtained by naively applying the traveling salesman algorithm to the semantic association graph. By default, the resulting list would have been tied to an arbitrary starting point even though empirically observed starting points across participants and trials were largely restricted to the first (23.8%) and the last (13.8%) object position. Thus, we actually conducted constrained runs of the algorithm to obtain two reference lists, one starting with the first and another one starting with the last object/memory item. The resulting semantic clustering indices were averaged within trials and participants.

In HOA, the resulting values ranged between 1.3 in learning trial one and 4.0 in sdfr. The YA obtained values between 1.2 as in learning trial one and 3.6 in the long delay-free recall (ldfr). Numerically, HOA was apparently superior in using semantic strategies. Nevertheless, the Wilcoxon rank sum tests failed to indicate substantial group differences. As Table 2 summarizes, median difference effect sizes were largely small even though they seemed somewhat more substantial as compared with those from the serial clustering indices. At the level of short delay and long delay-free recall, there was at least a tendency indicating a higher use of semantic strategies in HOA as compared with YA.

**Table 2:** The median and interquartile range of the semantic clustering index.

	HOA		YA		Effect size^a^	p^b^
	*N* = 18		*N* = 30			
	Median	IQR	Median	IQR		
trial1	1.28	[0.45;1.79]	1.17	[0.35;2.07]	0.07	0.639
trial2	3.36	[2.34;4.91]	2.49	[1.39;4.16]	0.21	0.148
trial3	3.74	[3.00;4.95]	2.97	[1.42;4.11]	0.18	0.217
sdfr	3.98	[2.68;4.66]	2.88	[1.70;3.89]	0.28	0.061
ldfr	3.65	[3.11;5.40]	3.60	[1.36;4.29]	0.27	0.067

a An effect size derived from the Wilcoxon z [z/sqrt(n)].

b Wilcoxon rank sum test.

HOA, healthy older adults; YA, young adults; IQR, interquartile range.

After the square root transformed the dependent variable, the modeling process resulted in a mixed model with random intercept and random slope (conditional *R*2 = 0.475). We obtained three fixed main effects extending on the group (F1, 46 = 5.5, *p* = 0.023), the log-transformed slope (trial; F1, 56.6 = 45.5, *p* < 0.001), and a sinusoidal correction term for slope (F1, 143 = 11.6, *p* < 0.001). There was neither an effect of demographic variables, general executive capacity, nor intelligence. The slope main effect indicated an average increase of 0.6 units per trial (*t* = 6.7, *df* = 190, *p* < 0.001). Because of its polynomial nature, the slope was numerically somewhat larger in HOA (0.63) than in YA (0.58). The semantic clustering indices increased in a nearly stepwise fashion across learning and recall trials. A major increase was observed between the first and the second learning trial and between sdfr and ldfr. The group’s main effect indicated the superior use of semantic strategies in HOA across all learning and recall trials by a difference of 0.63 units (*t* = −2.35, *df* = 46, *p* = 0.023). As with the modeling of the serial clustering indices, the marginal *R*2 was rather small (0.196).

### 3.2. Differences and similarities of encoding as determined by the most important associations

#### 3.2.1. Structural properties

As outlined in the Section 2, we created an association graph containing only the most important associations from both the HOA and the YA. The total number of retained edges, average vertex degree, clustering coefficient, and median edge weight differed only marginally in the graphs of the respective group (see Table 3). Thus, pruning might apparently have resulted in graphs of comparable complexity and associative strength. However, the small-world index *σ* (*p* < 0.001) and mean path length (*p* = 0.006) differed significantly between the graphs in favor of the HOA. Thus, the small-worldness of the YA and HOA graphs resulted in 1.75- and 2.39-fold higher values than expected by the vertex count of the respective networks. *σ* was based on the sampling of 3,000 random graphs per group. Taking into account that pruning retained only 6.4% of all pairwise associations in both the HOA and the YA graphs, the encoding strategies adopted by the HOA might nevertheless have resulted in associations of superior efficacy.

**Table 3:** Mean and standard deviations from major graph indices.

Variable	Graph HOA		Graph YA		Effect size^b^	*p*
	Mean	sd/%^a^	Mean	sd/%^a^		
Edges retained	64	6.4%	59	6.4%	0.00	1.000
Weight	0.11	0.03	0.11	0.03	0.02	0.847
Serial clustering	12	18%	23	39%	0.21	0.016
Vertex degree	2.78	1.20	2.57	1.00	0.12	0.399
Clustering coef.	0.17	0.28	0.12	0.24	0.18	0.230
Path length	0.51	0.12	0.56	0.10	0.40	0.006
Small world s	2.17	0.20	1.59	0.24	1.65	<0.001

a Where appropriate frequencies are shown. This is indicated explicitly by a % sign. Otherwise, the column refers to standard deviations.

b Effect size derived from the Wilcoxon signed rank *z* [z/sqrt(n)] and the Chi-square phi.

HOA, healthy older adults; YA, young adults; sd, standard deviation.

#### 3.2.2. Common subgraphs or cliques

**Figure 1:**
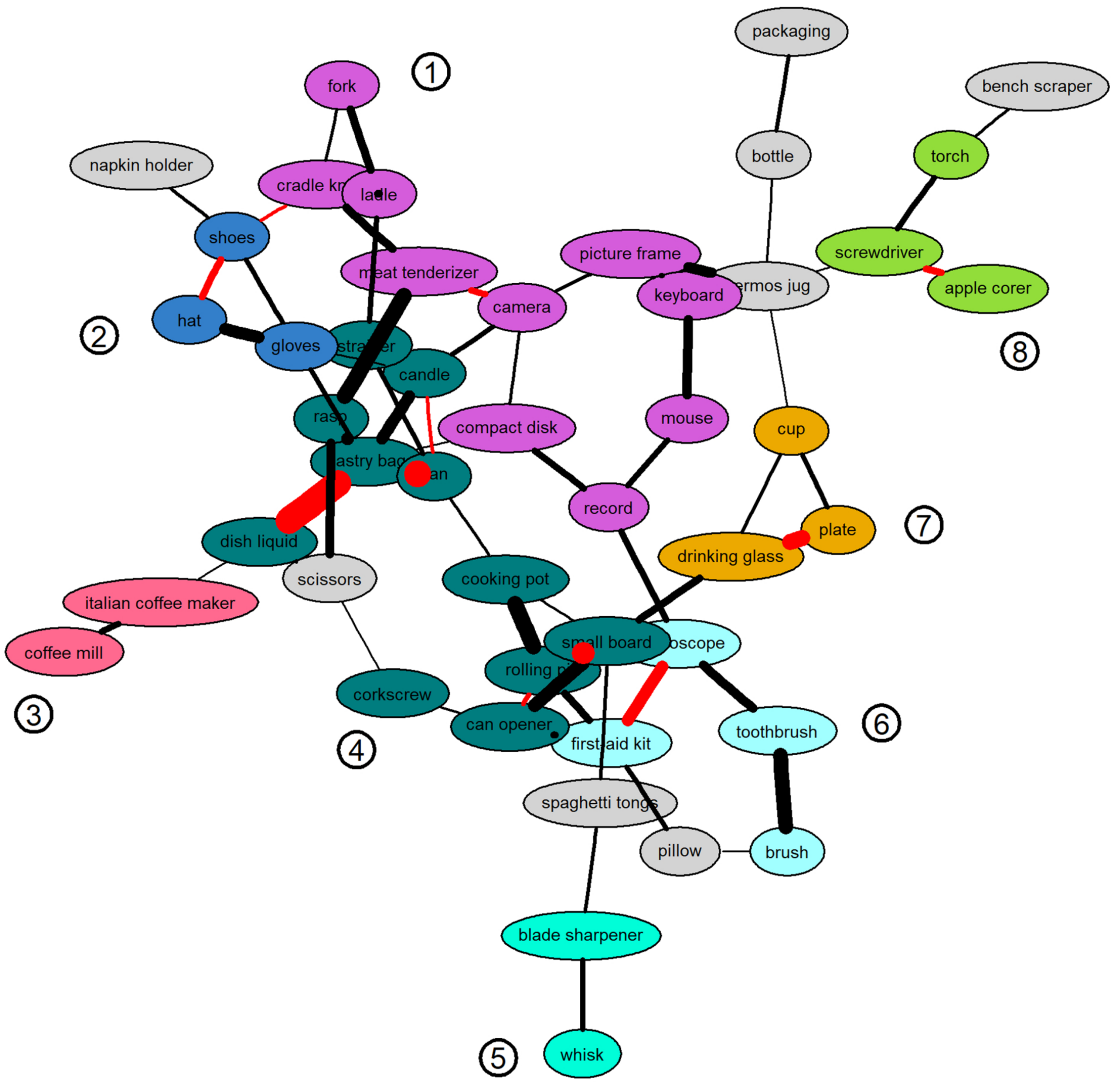
Memory association graph of objects encoded across five trials of free recall in healthy older adults (HOA). Note: Vertex color denotes subgraphs (numbered cliques) that both the HOA and the YA graphs have in common. Cliques are consistent with the following semantic grouping principles: cutlery hyponyms and media devices bridged by a serial association (1), clothes (2), coffee tools (3), objects from then primacy and recency portion (4), kitchen tools made of the same material (5), medical instruments and one hyperonym (6), dinnerware (7), and tools or shape (8). Red edges indicate consecutive serial list positions and edge width indicates a higher strength of association (smaller likelihood) as the width increases. Grayed vertices denote unassigned objects.

As shown in Figure 1, the graphs each contained 46 vertices according to all objects presented during the VR memory examination (see Figure 1). The reader might want to refer to supplementary materials (section 3) for a complementary visualization of the adjacency matrix. Vertex coloring was derived from the result of an intersection graph. Same-color vertices indicated cliques or connected components observed in both the HOA and the YA graphs. Thus, these might indicate converging encoding strategies for certain subsets of objects. In fact, there was no mean likelihood difference between within-clique edges (*F*1, 47 = 0.32; *p* = 0.575). However, between within-clique and between-clique edges, the association likelihood was significantly (*F*1, 121 = 14.1, *p* < 0.001) lower in edges belonging to connected components (mean = 0.10, sd = 0.03) compared to those that spanned connected components (mean = 0.13, sd = 0.03). In total, there were eight such cliques with at least two connected vertices. As closer inspection suggests, the cliques might reflect different semantic organization principles, such as categorical grouping (e.g., coffee mill and Italian coffee maker), grouping according to primacy and recency serial position effects (refer to Supplementary Materials for a complete list of objects in the presentation order), functional similarity (e.g., can opener and corkscrew), or even shape (e.g., apple corer, screwdriver, and torch). Especially, the larger subgraphs (> 3 vertices) were composed of chains of associations extended by hyponyms and hypernyms or semantically unrelated categories bridged by the exploitation of serial associations functioning as the interface (see Figure 1, clique 1). Since defined as object inherent properties, context specificity (novelty; Chi^2^ = 21.7, *p* < 0.001) and spatial location (Chi^2^ = 25.3, *p* = 0.017) were significantly associated with the cliques and, thus, definitely subserved memory organization in both groups. Additional but less obvious organizational principles might have been applied.

#### 3.2.3. Edge properties and validity

The red edge color indicates associations based on consecutively presented objects during the VR memory examination (serial position, see Figure 1). As shown in Table 3, a simple Fisher test for 2 × 2 contingency tables revealed a statistically significantly higher proportion of serially related edges regarding the YA (39%) as compared with the HOA graph (18%; *p* = 0.016). To determine the relationship between Lin’s semantic similarity measure and the association likelihoods, we conducted an analysis by regressing the likelihoods on Lin’s similarity measure, age groups, and serial clusters. Goodness-of-fit was maximized if seven objects from the primacy and three objects from the recency region were ignored (*R*2 = 0.15; *F* = 6.4; *df* = 2, 37; *p* = 0.003). The resulting model encompassed two main effects extending Lin’s semantic similarity measure (*F* = 8.7, *df* = 1, *p* = 0.004) and the serial clusters (*F* = 4.1, *df* = 1, *p* = 0.047). There was no effect on the age group. An increase of Lin’s measure by one standard deviation was associated with a decrease in mean likelihood by approximately 0.01 units (*t* = −3.2, *df* = 1, *p* = 0.002). Serial clustering contributed by further decreasing mean likelihood by a value of approximately 0.02 units (*t* = −2.0, *df* = 1, *p* = 0.047). Regression analysis suggested a primacy effect encompassing the first seven and a recency effect spanning the last three objects. In probing the first ten items from the participants’ memory protocols, a mean of 2.5 (sd 1.3) objects in YA and a mean of 1.4 (sd 0.6) objects from the primacy region in HOA were observed (*z* = −3.4, *p* < 0.001). A non-significantly different number of objects were observed for the recency region (YA: 1.1, sd = 0.6, HOA: 0.9, sd = 0.5, *z* = 1.1, *p* = 0.273).

## 4. Discussion

We could show that HOA made more intense use of semantic strategies in encoding various objects during an ecologically sound VR memory examination as compared to YA. These encoding strategies or memory organization principles were reflected by the serial order of object recall. The superior utilization of semantic strategies by the HOA pervaded all learning and delayed recall trials. However, since memory performance in both the HOA and YA was basically similar or almost identical as was previously shown by Pflueger et al. (2018), we hypothesized that we might find evidence for YA relying more on rote memory as compared to HOA. Clear evidence for rote memorization is the sequential learning of memory items in order of appearance. Thus, we expected the YA to reveal a greater proportion of serial clustering and extended serial position effects compared to HOA. A direct comparison of serial clustering between HOA and YA did not indicate overly superior use in YA. However, by focusing on the most important associations as determined by the object memory association graphs (networks), there was clear evidence of an increased serial encoding and a larger primacy effect in YA. The larger primacy effect was not only evident in the association graph approach but could be confirmed by examining individual memory protocols as well. Thus, there is at least partial evidence in support of our hypothesis regarding the YA.

It is proposed that essentially two factors may account for age-related cognitive changes across the lifespan. These two factors are executive capacity and crystallized abilities (Craik and Bialystok, 2006). While executive capacity is known to decline with age, crystallized abilities constantly grow or, at least, remain stable with advanced age. Memory performance in healthy older adults is known to be associated with the functional recruitment of additional frontal brain areas not typically recruited in younger adults (Cabeza et al., 2016) and the availability of viewer and less effective memory strategies (Lemaire, 2010). As opposed to the young adults’ memory performance, it does not only correlate with the measures of crystallized abilities but also with executive capacity (Bouazzaoui et al., 2013). Moreover, evidence suggests that 82% of age-related variance in the mental memory strategy use is accounted for by executive functions (Bouazzaoui et al., 2010). This pattern is thought to be due to preserved crystallized abilities, but yet diminished executive resources, and might at least partially reflect an additional compensatory effort in older adults in order to maintain a sufficiently high level of performance. However, in the current analysis, we found no effect of general executive capacity on either performance or semantic clustering. Thus, in accordance with our previously reported notion, we assume that the adoption of specific encoding and retrieval strategies in the VR memory examination is rather effortless and automatic, as opposed to the clinically deployed word list learning. Moreover, the lack of additionally activated executive resources is consistent with the idea that HOA does not have to apply compensatory strategies in order to generate effective memory organization strategies. Instead, the crystallized abilities alone may be sufficient. Thus, the observed superior semantic clustering in the HOA is possibly a consequence of the age-related increase in these abilities.

As a consequence of its ecological validity, the VR memory examination draws on an enhanced coding model as computational considerations and empirical evidence on object memory suggest (Brady et al., 2011). The coding model is assumed to be a “multidimensional feature space” that can be conceived of as a hierarchical tree-like structure representing conceptual category-specific features at its top node (root) and rather perceptual category-general features as its leaves. According to the conceptual structure approach (CSA), the categories and domains of knowledge are not explicitly represented. Instead, object representations are feature-based downstream of the ventral temporal cortex (Taylor et al., 2011). Current evidence suggests that the statistical properties of those features predict successful retrieval from object memory regardless of lexical or visual cues (Hovhannisyan et al., 2021). This gives rise to the wealth of different encoding strategies such that the superior crystallized abilities in older adults might sufficiently compensate for an age-related decline in various other and specific cognitive domains.

By obtaining a statistically significant negative association between the likelihood from the association networks and Lin’s semantic similarity measure (derived from the GermaNet database), we could convincingly demonstrate the construct validity of our association graph approach. The likelihoods reflected, at least partially, semantic principles, which might be a consequence of the use of semantic encoding strategies. The networks differed structurally in terms of connectivity but revealed joint and coherent clusters (cliques) of semantic entities, which are likely to reflect converging encoding strategies in both the HOA and the YA. The cliques resulted from an intersection graph analysis, and coherence was demonstrated by lower within-clique than between-clique association likelihood. We showed that the cliques were associated with spatial location encoding and context specificity of the encoded objects. Other encoding principles are applied as well. For instance, a particular cluster was almost exclusively composed of objects belonging either to the primacy or to the recency region of the presented items. The visual inspection of the networks suggested additional memory organization principles. The clusters might, therefore, have reflected the principles of categorical and functional similarity or even similarity according to color, shape, or other more basic and not too obvious (sensory) properties. A striking observation is consistent with the idea that serially related objects served as an interface between semantically unrelated sub-clusters within the same clique or between different cliques. Thus, there might be a multitude of different encoding mechanisms, as also suggested by the conceptual structure account (Taylor et al., 2011) and even integrating serial and semantic strategies, which were beyond the scope of the current data analysis.

In addition to similarities, the network analysis also revealed structural differences regarding the connectivity of the respective networks. We could show that the object memory association networks in both healthy older adults and young adults exhibit small-world properties. However, the small-worldness of the older adult’s association network was superior compared to the young adult’s network. Small-worldness indicates efficient information segregation and integration with low energy and wiring costs. Translated to the object memory association networks, this suggests high efficiency with which different nodes in different parts of the network might be accessed and retrieved. Irrespective of their originating domain or other topological properties, small-world properties can be found in a multitude of social, information, technological, or biological networks (Humphries and Gurney, 2008). The same is true regarding the human brain’s structural and functional networks. Topological properties from functional brain networks cannot easily be inferred from structural networks and vice versa, even though a close relationship between the two might exist. Functional brain networks change in configuration depending on task demands at a time scale of seconds to minutes and even spontaneously fluctuate in the resting state without external task demands. This fluctuating configuration might reflect mental state shifts (Liao et al., 2017). It should be noted that the object memory association networks definitely do not correspond to a single mental state since the structural properties of these networks were amalgamated across multiple trials of encoding and retrieval and multiple participants. Emanating from rather diffuse initial organization strategies contingent with a higher proportion of random associations, one might expect consolidation to more solid strategies with advanced learning. Thus, we assume that the observed object memory association networks are more likely to reflect memory organization principles according to advanced and consolidated learning than simply and indiscriminately reflecting a mere mean across all trials and participants. In addition, this would explain the considerably low proportion of preserved associations compared to the respective unpruned networks. In general, the evidence of our analysis clearly indicates that the observed structural properties of the association networks were not random. Instead, they likely reflect frequently used and closely resembling encoding strategies across learning trials and participants. Thus, if one grants a higher variety and diversity of semantic strategy use in the HOA as compared to the YA (as we argue), then it is highly likely that the resulting association networks might exhibit a more complex connectivity pattern in terms of the small-world property. Thus, we conclude that the small-worldness of a conjoint object memory association network reflects the extent to which effective semantic strategies diverge between participants as a result of a superior semantic memory organization.

As pointed out in a recent review, multiple psychopathological and neuropathological conditions are associated with an altered small-world organization of functional and structural brain networks. There is evidence according to which functional and structural human brain networks in patients suffering from Alzheimer’s disease tend to show a rather regular configuration with a decreased global efficacy of information transmission. Therefore, it is not further astonishing that irregularities in small-world attributes are discussed as potential biomarkers for early detection, diagnosis, and treatment (Liao et al., 2017).

One striking and marked issue concerns the effect sizes of most of the reported results. With few exceptions, we mostly observe small to very small effects. This may raise concerns as to whether our results are scientifically relevant. However, the aim of the recurring data analysis was to show that the primary result of the VR memory examination (almost equal memory performance in a sample of HOA and YA) was due to ecological validity rather than a methodological artifact. Because of the coherent results in support of our hypotheses, we claim to have reasonable arguments in favor of this view even though the observed effects are rather small. Semantic relationships in terms of the GermaNet database are defined along the dimensions of homonymy and meronymy. However, a plethora of other semantic relationships or associative techniques is conceivable as a visual inspection of the object memory association networks and the conceptual structure account (Taylor et al., 2011; Hovhannisyan et al., 2021) suggests. Thus, it is evident that from Lin’s semantic similarity measure or from the semantic clustering index, only marginal proportions of variance might be explained.

Association networks derived from single learning, and recall trials could reveal how a specific network grows and its structure unfolds as learning proceeds. This would allow us to study and describe learning with graph theoretical or network topological methods or to investigate whether the association networks follow a nonlinear dynamic trajectory. In general, this could provide a new toolbox for examining episodic memory in healthy and pathological aging. However, it would require much larger samples than the ones we analyzed.

### 4.1. Conclusion

In summary, we could show that an ecologically valid memory examination by means of virtual reality is associated with a reduced need for compensatory efforts in healthy older adults. This lack of compensatory requirements may be due to an enhanced coding model for everyday objects. Thus, older adults can make comprehensive use of superior crystallized abilities in encoding everyday objects into episodic memory. The superior crystallized abilities might be sufficient to counteract an age-related decline in various other and specific cognitive domains.

## Data availability statement

The original contributions presented in the study are included in the article/Supplementary material, further inquiries can be directed to the corresponding author.

## Ethics statement

The studies involving human participants were reviewed and approved by Ethikkommission beider Basel EK: 256/12. The patients/participants provided their written informed consent to participate in this study.

## Author contributions

MP conceived, designed, and supervised the study, performed the data analysis, and wrote the manuscript. RM, R-DS, and TL helped to supervise the project and contributed to the manuscript. MG provided the technical infrastructure and the indispensable VR environment. All authors discussed the results and contributed to the final manuscript.

## Conflict of interest

The authors declare that the research was conducted in the absence of any commercial or financial relationships that could be construed as a potential conflict of interest.

## Publisher’s note

All claims expressed in this article are solely those of the authors and do not necessarily represent those of their affiliated organizations, or those of the publisher, the editors and the reviewers. Any product that may be evaluated in this article, or claim that may be made by its manufacturer, is not guaranteed or endorsed by the publisher.
