# Supplementary material for: Encoding of everyday objects in older adults: Episodic memory assessment in virtual reality
Source: Front Aging Neurosci. 2023 Mar 13;15:1100057. doi: 10.3389/fnagi.2023.1100057 (PMC10040840; doi:10.3389/fnagi.2023.1100057)
Supplement: Supplementary file 2 [file Data_Sheet_2.docx]

Supplementary Materials

# Sample Description

The study encompassed 30 young and 18 older participants. Demographic data are shown in Table [1](#tab:tab_desc). In addition to the VR memory examination, the participants rated the weekly use of the computer (amount of time) and the degree of immersion felt during the examination. Degree of immersion was scored on a 5-point Likert scale from 1 (felt not very realistic) to 5 (felt extremely realistic). There were no statistical differences regarding education, gender, and feeling of realism between the HOA and the YA. Median age was 44.5 years higher in advanced agers compared with the YA, and the HOA spent approximately half of the time on computers per week (10.8 hr) than YA did (20.7 hr). Verbal intelligence was significantly higher in HOA, compared with YA.

**Table 1:** Mean, Standard Deviation, and Interquartile Range of Demographic Variables, Computer Use, and Intelligence Stratified by Groups of Young Adults and Healthy Older Adults (55+)

|  | HOA | (N = 18) |  | YA | (N = 30) |  |  |  |
| --- | --- | --- | --- | --- | --- | --- | --- | --- |
| Variables | M | (SD) | IQR | M | (SD) | IQR | effect | P |
|  |  |  |  |  |  |  | Size |  |
| age (yrs) | 69.7 | (7.0) | 8.8 | 27.7 | (8.3) | 4.5 | 0.83 | <0.001 |
| education (yrs) | 15.8 | (5.5) | 7.8 | 16.4 | (2.6) | 3.0 | 0.09 | 0.664 |
| gender (female, %) | 9 | (50.0%) | NA | 23 | (76.7%) | NA | 0.23 | 0.114 |
| computer use (hrs/week) | 10.8 | (8.8) | 7.0 | 20.7 | (12.7) | 23.8 | 0.38 | 0.009 |
| feeling of realism | 3.5 | (1.2) | 1.0 | 3.9 | (0.9) | 1.8 | 0.15 | 0.310 |
| non-verbal IQ | 112 | (14.4) | 15.8 | 119 | (8.6) | 11.3 | 0.28 | 0.055 |
| verbal IQ | 122 | (14.6) | 18.0 | 113 | (14.6) | 21.5 | 0.30 | 0.046 |
| Executive capacity | -0.002 | (1.3) | 1.8 | 0.001 | (0.9) | 1.1 | 0.80 | <0.001 |

# Generating an association graph from memory protocols

A graph is defined as a collection of vertices (or interchangeably nodes) and edges. It is a structure of objects (vertices), which are at least partially related to one another (edges). Vertices that are directly connected by one edge are called neighbors. A graph can be directed or undirected. In a directed graph edges have arrows, i.e. relations between the vertices are non-reciprocated. In an undirected graph all relations are reciprocated, thus, edges have no arrows. Edges may be weighted or unweighted. If a graph contains unweighted edges, then all edges have the same weight of one. If it contains weighted edges, then the graph is called a network with relations that differ in strength. A graph might be connected or unconnected. In a connected graph, every vertex is principally accessible from any other vertex through a series of edges (path) within the graph. If edges are removed (pruned), a connected graph might decay in groups of unconnected cliques (components, or subgraphs) and, thus, become an unconnected graph. A graph may be represented by an adjacency matrix, i.e. a square matrix where the elements determine whether a pair of vertices are adjacent or neighbors (i.e. have one edge between them). An undirected graph is represented by an adjacency matrix, which is symmetric with respect to the main diagonal or which is reduced to a triangular matrix (see figure [1](#fig:adj_matrx)). If the graph is weighted, the elements of the adjacency matrix are reflecting these weights.

<insert fig1suppl about here>

**Figure 3:** The adjacency matrix (left) of an unconnected and undirected graph (right)

An adjacency matrix can be produced from memory protocols, if the memory protocols encode serial list positions of the objects during free recall. The following example of a memory protocol recorded during free recall of learning trial one might illustrate this in more detail:

Trial1: 1 41 27 42 12 13 23 24 10 36 16 6 30 44 29 5 33 38

Each consecutive pair of values increments the respective element of the adjacency matrix by a value of one. Thus, the first pair {1 41} adds to the first row and 41th column, the second {41 27} to the 41th row and the 27th column, etc. This may be repeated for all learning trials, short, and long delay free recall and for all participants in a group of interest. However, the resulting weights (or absolute frequencies) cannot be compared between groups of different sample sizes. Moreover, we do not know how to interpret these weights with respect to chance. Therefore, we calculated the expected frequency for each cell in the adjacency matrix and divided it by the observed frequency (weight). This yielded a likelihood, where a small value indicated a low probability of association by chance (see figure 2). Thus, the most important associations were those that were characterized by the smallest likelihood values. Since we assume associations between any two objects to operate bidirectionally, we were interested in an undirected association graph. Consequently, we collapsed all symmetric node pairs into the upper triangular matrix by addition.

<insert fig2suppl about here>

**Figure 2:** The relationship between the observed F_obs_ and the expected frequencies F_exp_. Expected frequencies presume non-contingency (i.e. no relationship) between the columns and the rows of a frequency table (i.e. the vertices of a graph). It is calculated as the product of the row and column sums divided by the total number of observations (depends on group sample size). As to yield association likelihoods, the expected frequencies were divided by the observed frequencies. Thus, a likelihood < 1 between two nodes indicates an association, which is more often observed than expected. Since nominator and denominator depend on the same sample size, the likelihoods are also comparable between groups of a different sample size.

Since we were merely interested in the most important associations, we pruned the graph such that less important edges were progressively removed as long as the graph remained connected. Figure [3](#fig:assoc_grph_ya) shows the pruned object memory association graph (network) of the YA.

<insert fig3suppl about here>

**Figure 3:** Memory association graph of objects encoded across five trials of free recall in YA. See Figure 1 in the manuscript for details.

An association graph can be characterized in terms of its complexity and associative strength. Since the graphs, which were subject to our analysis, have the same number of vertices, we defined complexity in terms of the number of edges and the average vertex degree, i.e. the mean number of neighbors or adjacent vertices per vertex. Closely related to this is the graphs' clustering coefficient (see equation 1 & 2). The clustering coefficient measures the extent to which the neighbors of a vertex are also interconnected. It is calculated as the ratio of the observed number of edges between the neighbors of a vertex and the number of maximal possible edges between them. The mean or global clustering coefficient of a graph might range from 0 (e.g. if the graph is degenerated to an ensemble of unconnected nodes) to 1 (complete graph, i.e. every pair of vertices is connected by an edge). Complex networks and notably small-world networks are defined by a high normalized clustering coefficient and a low mean path length (the mean length of any shortest path between each pair of vertices, see equation 3). Thus, small-world networks are characterized by low wiring costs and both a high local and global interconnectivity. As a consequence the system dynamics (i.e. efficiency of activation spreading) is much higher than in regular networks like simple lattices (Watts and Strogatz, 1998). Small-world-ness is not an all-or-none property, but might occur to different degrees in different networks (Humphries and Gurney, 2008). In terms of the object memory association graphs, this means that recall efficacy (or recall probability) of an arbitrary object increases as the small-world-ness of the association network increases. It can be calculated as the ratio of the networks' normalized clustering coefficient and mean path length. Normalization is achieved by deriving the same indices from randomly sampled graphs with same node-edge count and same weights and dividing the graphs' coefficients by these values (see equation 4). Finally, a small-world index σ > 1 indicates small-world-ness.

$C_{i}=\frac{number of closed triples connected to node i}{number of triples connected to node i} (1)$

$$C=\frac{1}{N}\sum_{i=1}^{N} C_{i} (2)$$

$$L=\frac{2}{N(N-1)}\sum_{i\neq j} d_{ij} (3)$$

$$\sigma=\frac{\frac{C}{C_{r}}}{\frac{L}{L_{r}}} (4)$$

where C denotes the clustering coefficient, N is the number of nodes in a graph, and L is mean path length, d_ij_ denotes the shortest path length between nodes i and j. Note, the subscript r indicates averaged indices based on multiple randomly generated graphs with same node-edge count.

Actually, both the small-world-ness σ (5) and the number of edges M (6) in a given graph are log-linearly proportional to the number of nodes (Humphries and Gurney, 2008). Therefore, this allows indexing a given network as above or below expected small-world-ness.

$$\sigma=0.023N^{0.96} (5)$$

$$M=2.46N^{1.06} (6)$$

## Small world networks

A network is said to have small-world properties if it is characterized by clusters of high local inter-connectivity and only spars remote connections between those clusters. Even though remote connections exist rather rarely, the dynamics of such a network is quite remarkable. Activity within one cluster soon reaches remote clusters and finally spreads exponentially across the entire network. It has been shown that social systems behave like small world networks. In an experiment by Stanley Milgram 296 arbitrarily selected individuals in Nebraska and Boston were asked to generate an acquaintance chain to an anonymous target person in Massachusetts (Travers and Milgram, 1977). A document was handed over that contained rather sparse information about the target person and a set of instructions of how the goal might be achieved. Probably the most important was not to attempt directly to contact the target person, if he or she is not personally known. Instead, the participants were instructed to mail to a personal acquaintance, who is more likely to know the target person. Finally, the document contained a roster on which each person should leave his or her name. Thus, the experimenters could easily retrace the stations the mail went through. As a result, 64 mails actually reached the target person with a mean number of 5.2 intermediaries (recipients between start and end). Milgram concluded that this surprisingly small number of intermediaries might connect any two persons around the globe (remember, small-world networks have a low mean path length between any two vertices). This is where the term small-world network was coined. Because any two persons who meet each other by chance and who might recognize a common friend or acquaintance in a remote part of the world usually come to the conclusion that the world must be small.

## Serial and semantic clustering

Serial and semantic clustering is a well-known and widely appreciated concept in the assessment of verbal learning which in clinical settings was initially introduced by the California Verbal Learning Test (Delis et al., 1984). The idea that organizational strategies might facilitate the later free recall is undisputed. In general, semantic strategies are viewed as superior over serial strategies. Serial strategies are conceived of as reflecting effects of the immediate experience with the word list such as serial position recency and primacy effects. Whereas semantic strategies emerge from reorganizing words according to semantic principles and, therefore, might reflect a subjectively more meaningful and concise entity to the individual. The CVLT provides two indices, which indicate the degree of how those strategies might have been used during recall of the word list. Thereby, the consecutive occurrence of any two words according to their adjacent list position increases the serial cluster score by a value of one. The analog is true, whenever two words of the same semantic category are consecutively recalled. Then the semantic cluster score is incremented by a value of one. The rationale of this scoring scheme is evident. However, the adjustment from clustering by chance appears somewhat more convoluted. As opposed to the first edition, the second and revised edition of the CVLT adopts list based clustering indices (LBC). The list based adjustment assumes that the number of clusters expected by chance only depends on list length and not on the amount of items or words retrieved for recall. There is both a theoretical and an empirical argument in favor of this perspective (Stricker et al., 2002). In general, the LBCs are expressed as follows: LBC = LBC_OBS_ - LBC_EXP_, where LBC_OBS_ corresponds to the observed and scored clusters and LBC_EXP_ equates to expected and calculated clusters by chance.

### The expected serial clusters

Provided we use an N-item word/object list, then the probability of obtaining a serial cluster by arbitrarily recalling any two items in succession is exactly 1/N. If r items are recalled, then there are r-1 opportunities of obtaining a serial cluster by chance. Thus, the serial LBC_EXP_ equates to LBC_EXP_ = (r-1)/N. No matter how many items are recalled, the probability of obtaining a serial cluster by chance always remains below 1. Things become somewhat more complicated, if the constraints of what applies to serial clusters are relaxed. If bidirectional recalls were accepted, then there were exactly two opportunities of obtaining a serial cluster by chance. Thus, the above equation would be multiplied by 2. Even more relaxed definitions of what applies to a serial cluster are conceivable. Suppose, the recall of any two items within a certain boundary (say < 3) of the serial position would be viewed as a cluster. This would not pose a serious problem either since it could be handled according to the same logical principles. During the VR memory examination, the objects were presented in following order:

dish liquid, pastry bag, pan, candle, rasp, strainer, scissors, gloves, meat tenderizer, camera, Italian coffee maker, hat, shoes, cradle knife, packaging, compact disk, ladle, corkscrew, napkin holder, thermos jug, fork, picture frame, coffee mill, keyboard, bottle, bench scraper, cup, pillow, screwdriver, apple corer, torch, blade sharpener, mouse, whisk, brush, record, toothbrush, spaghetti tongs, stethoscope, first-aid kit, plate, drinking glass, cooking pot, can opener, rolling pin, small board

### The observed and expected semantic clusters

Semantic LBC_OBS_ as well as LBC_EXP_ cannot that easily be derived from the CVLT by analogy with the expected serial clusters since the objects/memory items were not selected according to a predefined semantic category. Therefore, an alternative post-hoc method was required as described in the appropriate methods section. The application of the traveling salesman algorithm resulted in a chain of objects characterized by a maximized pairwise semantic similarity. However, the solution to this problem is not trivial since in principle there are 46! (factorial) paths to inspect. In fact, the traveling salesman problem (TSP) is NP-complete. Fortunately, there are a number of algorithms providing near-optimal solutions to the problem at hand. Actually, we used the Concorde TSP solver, which is currently the best-performing exact TSP solver. It has previously been used to solve the largest non-trivial TSP instances for which provably optimal solutions are known. Concorde is based primarily on a complex branch & cut algorithm that uses a multitude of heuristic mechanisms to achieve good performance on a wide range of TSP instances (Hoos and Stützle, 2014).

Consecutively recalled items that were adjacently found in the semantic reference list, irrespective of order, incremented the semantic LBC_OBS_ by a value of one. Since we recurrently dealt with serial positions (although this time referring to the semantic reference list) the problem of determining an appropriate number of expected semantic clusters by chance reduced to the rational of finding an equation for expected serial clustering. Because scoring occurred bidirectionally, the number of expected semantic clusters by chance equated to LBC_EXP_ = 2*(r - 1)/N. Where r is the number of correctly recalled items and N denotes list length.

## GermaNet and semantic similarity measures

GermaNet (Hampel and Feldweg, 1997; Henrich and Hinrichs, 2010) is a lexical-semantic net that relates German nouns, verbs, and adjectives semantically by grouping lexical units that express the same concept into synsets and by defining semantic relations between these synsets. GermaNet has much in common with the English WordNet®. It can be viewed as an on-line thesaurus or a lightweight ontology.

GermaNet has been developed and maintained within various projects at the Division of General and Computational Linguistics, at the University of Tübingen (Germany) since 1997. Refer to the GermaNet Website (<http://www.sfs.uni-tuebingen.de/GermaNet/>) as to obtain information that is more detailed.

The database stores all its synsets (128100) in a tree-like semantic hierarchy from the most general one (hyperonymy), which constitutes the root of the tree, down to the most specific ones (hyponymy), which constitute its leaves. These hyponym-hypernym relations are also called ISA relations, since they all follow the same pattern of, e.g. an oak (hyponym) *is a* tree (hypernym). Additionally, synsets might be interrelated by part-whole patterns (meronymy), like e.g. glass is part of a window. As opposed to the vertical ISA relations, this allows for horizontal relations between pairs of synsets.

We used the GermaNet version 13.0 as to obtain semantic similarity metrics related to any two objects presented during the VR memory examination. All objects were translated to the corresponding synsets and pairwise semantic similarity measures were determined according to (Lin, 1998):

$$sim_{L}\left( c_{1},c_{2} \right)=\frac{2\times logp(lso(c_{1},c_{2})}{\log p\left( c_{1} \right)+\log p\left( c_{2} \right)} \left( 7 \right)$$

where *sim_L_* denotes Lin's semantic similarity measure, *c_1_* and *c_2_* are the respective synsets, *p* denotes the probability of obtaining an instance of a synset in a specific corpus (Henrich and Hinrichs, 2014), and *lso* denotes the lowest-superordinate (most specific common subsumer). If the probability of obtaining the *lso* corresponds to the mean probability of the respective synsets, then the semantic similarity of the synsets is maximal and its value amounts to one. However, if there is no *lso* at all (which is rather unlikely) or its probability to be obtained in a respective corpus converges towards zero, then the semantic similarity likewise converges towards zero.

# Adjacency matrices of the older and younger adults’ object memory networks

<insert Figure AdjOlder about here>

**Figure 4:** Adjacency matrix of the older adults object association network. The first object of the learning trials was “dish liquid”, the last one was the “small board”. The brighter the colors, the higher is the association likelihood between two objects. Note that the values are mirrored along the main diagonal. Especially dens are associations from the beginning and the end of the object list. These might reflect serial position effects. Most associations are regional and limited to a subset of items. Associations across large proportions of the object list are rare.

<insert Figure AdjYounger about here>

**Figure 5:** Adjacency Matrix of the younger adults’ object association network. The same principles hold: the brighter the color, the higher is the association likelihood between two objects. The primacy and recency areas are markedly more pronounced then in the older adults’ adjacency matrix. However, regional limitation is significantly more pronounced in the younger adults’ adjacency matrix. See text below for details.

In computing the orthogonal projection of every single association to the main diagonal of the matrix, the Euclidian distance of a respective association to the main diagonal can be calculated. Thus, we have a means to decide, whether one of the groups shows statistically relevant local constraints as implied by visual inspection. Local constrains refer to the distance between two associated objects regarding the previously determined presentation order during the learning trials, i.e. the number of interleaving objects between the presentation of the two (associated objects). Median Euclidian distance (number of interleaving objects) from the associations to the main diagonal in old adults is md = 2.83 objects (IQR=3.71 objects). With regard to young adults we observe md = 1.41 objects (IQR=2.83 objects), which is significantly lower (Wilcoxon W=2387, p ~ 0.01). The limited ability (intention) of the younger adults to establish associations across a larger subset of items compared to the older ones is consistent with the hypothesis that they more relay on serial strategies and, thus, are less likely to use semantic strategies.

# References

Delis, D.C., Kramer, J., Kaplan, E., Ober, B., 1984. California Verbal Learning Test. Test critiques 158.

Hampel, B., Feldweg, H., 1997. Germanet-a lexical-semantic net for german, in: Automatic Information Extraction and Building of Lexical Semantic Resources for NLP Applications.

Henrich, V., Hinrichs, E., 2014. Consistency of manual sense annotation and integration into the t{\"u}ba-d/z treebank.

Henrich, V., Hinrichs, E., 2010. GernEdiT-the GermaNet editing tool, in: Proceedings of the ACL 2010 System Demonstrations. pp. 19–24.

Hoos, H.H., Stützle, T., 2014. On the empirical scaling of run-time for finding optimal solutions to the travelling salesman problem. European Journal of Operational Research 238, 87–94. https://doi.org/10.1016/j.ejor.2014.03.042

Humphries, M.D., Gurney, K., 2008. Network ‘Small-World-Ness’: A Quantitative Method for Determining Canonical Network Equivalence. PLoS ONE 3, e0002051. https://doi.org/10.1371/journal.pone.0002051

Lin, D., 1998. An information-theoretic definition of similarity, in: Icml. pp. 296–304.

Stricker, J.L., Brown, G.G., Wixted, J., Baldo, J.V., Delis, D.C., 2002. New semantic and serial clustering indices for the California Verbal Learning Test–Second Edition: Background, rationale, and formulae. J Int Neuropsychol Soc 8, 425–435. https://doi.org/10.1017/S1355617702813224

Travers, J., Milgram, S., 1977. An Experimental Study of the Small World Problem, in: Social Networks. Elsevier, pp. 179–197. https://doi.org/10.1016/B978-0-12-442450-0.50018-3

Watts, D.J., Strogatz, S.H., 1998. Collective dynamics of ‘small-world’ networks. Nature 393, 440–442. https://doi.org/10.1038/30918
