# Supplementary figures and images for: Encoding of everyday objects in older adults: Episodic memory assessment in virtual reality
Source: Front Aging Neurosci. 2023 Mar 13;15:1100057. doi: 10.3389/fnagi.2023.1100057 (PMC10040840; doi:10.3389/fnagi.2023.1100057)

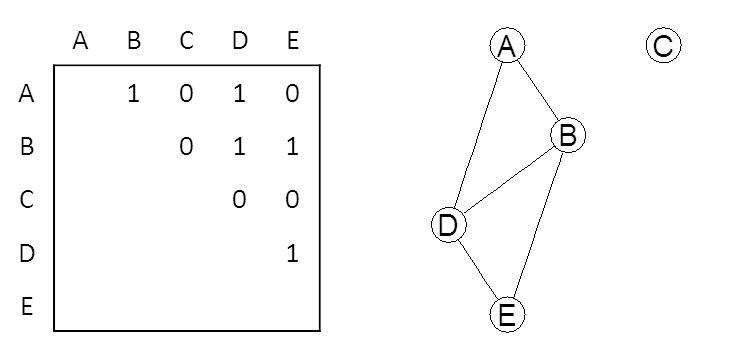

Supplement: Supplementary file 3 [file Image_1.JPEG]

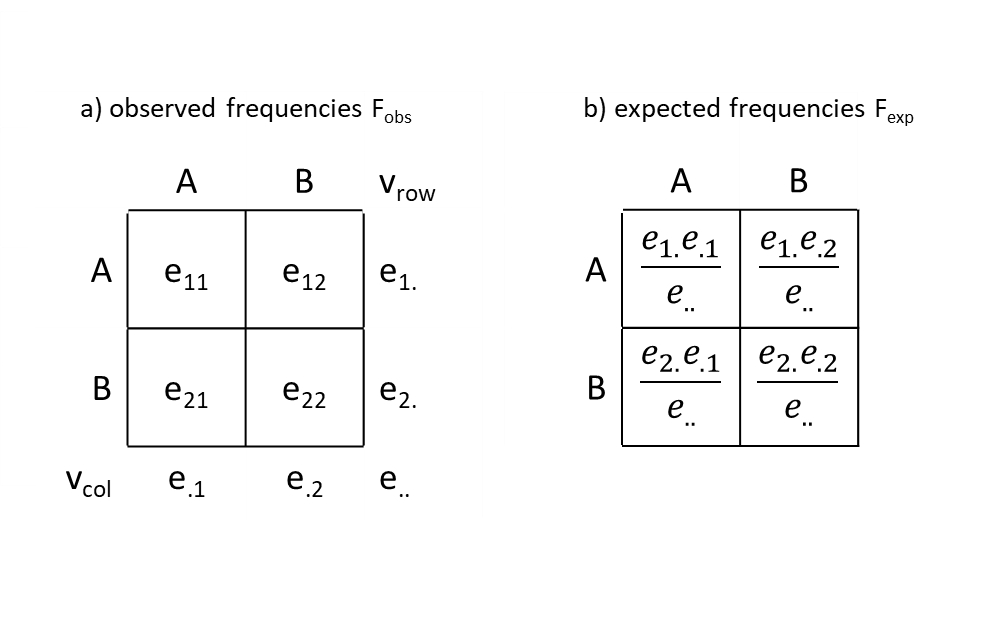

Supplement: Supplementary file 4 [file Image_2.JPEG]

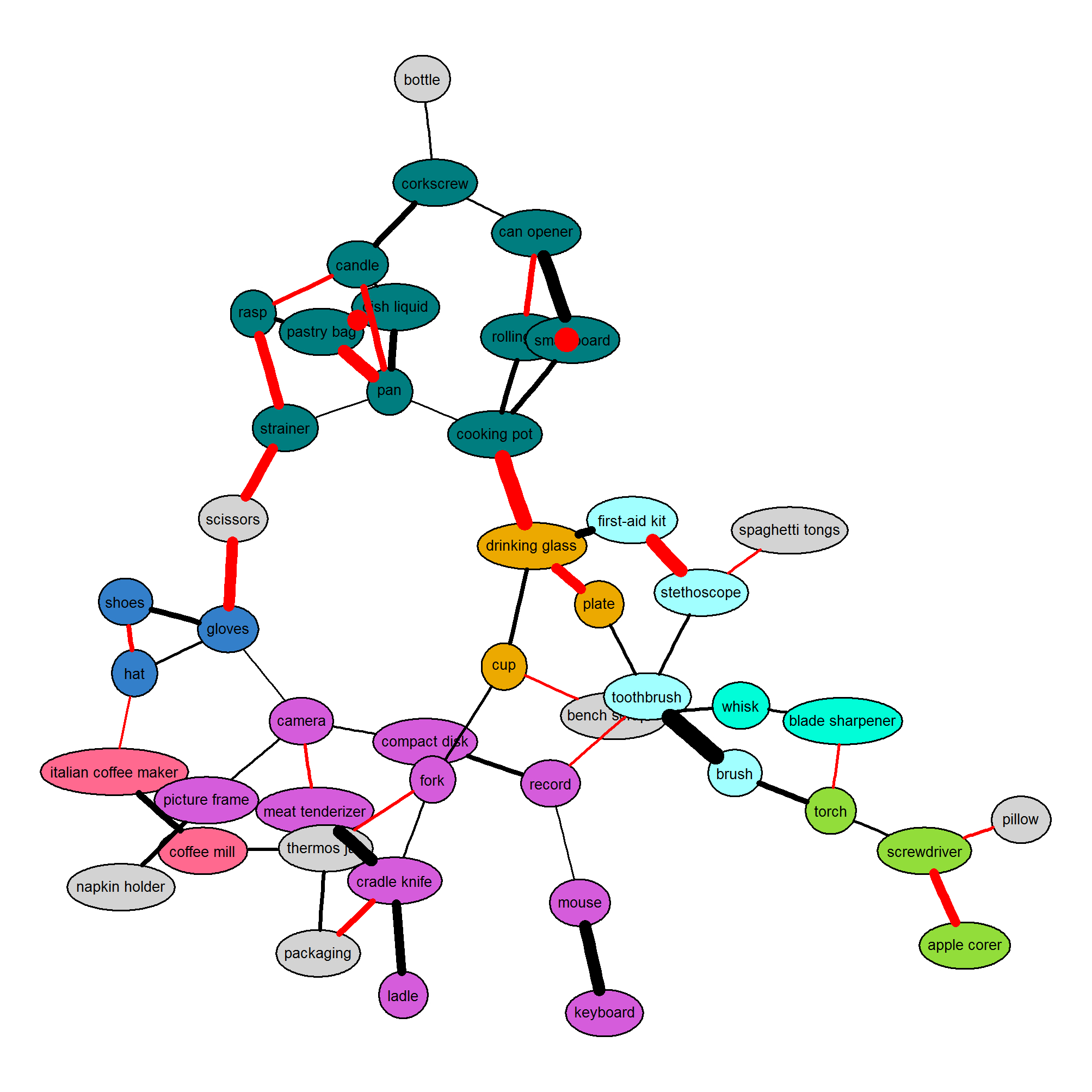

Supplement: Supplementary file 5 [file Image_3.JPEG]

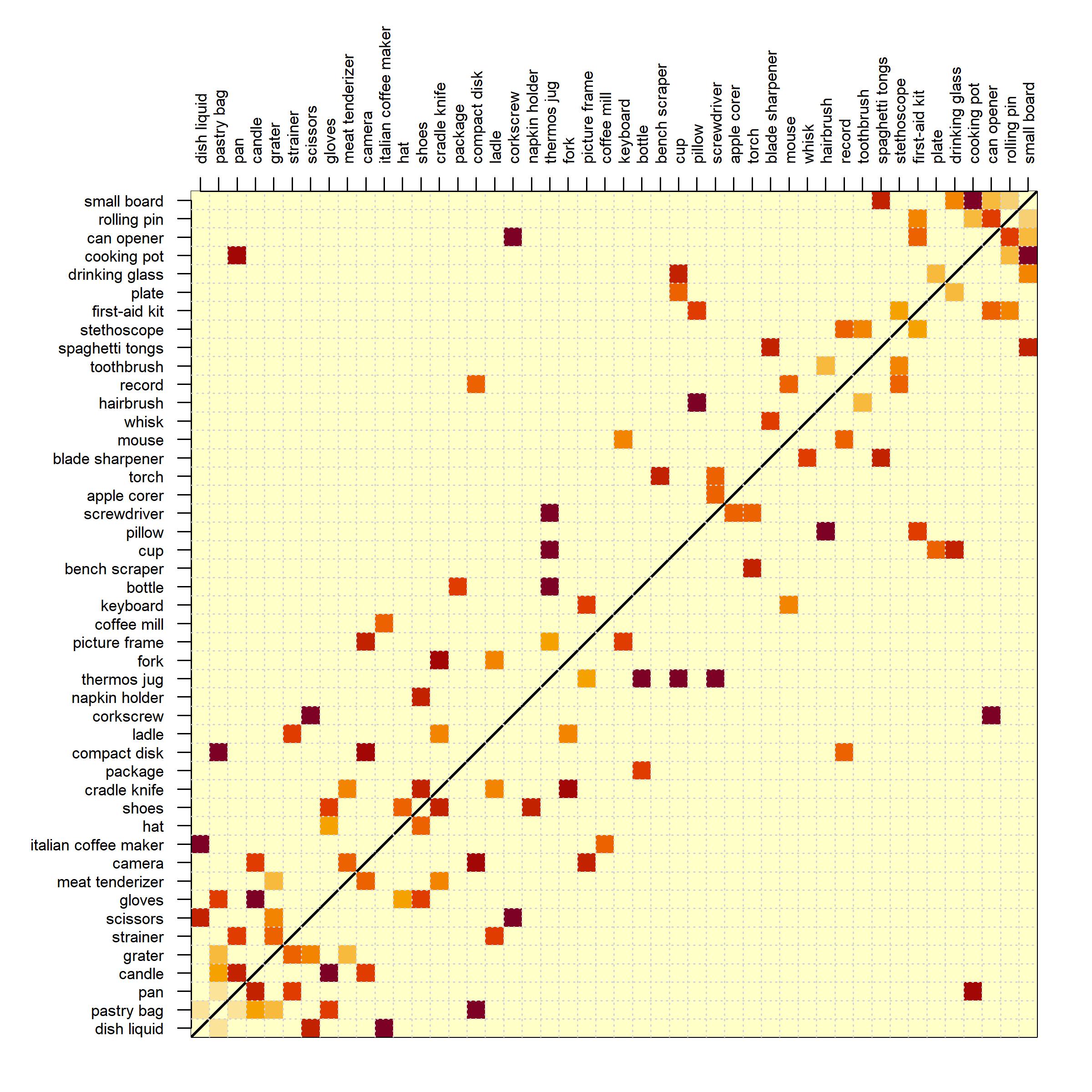

Supplement: Supplementary file 6 [file Image_4.JPEG]

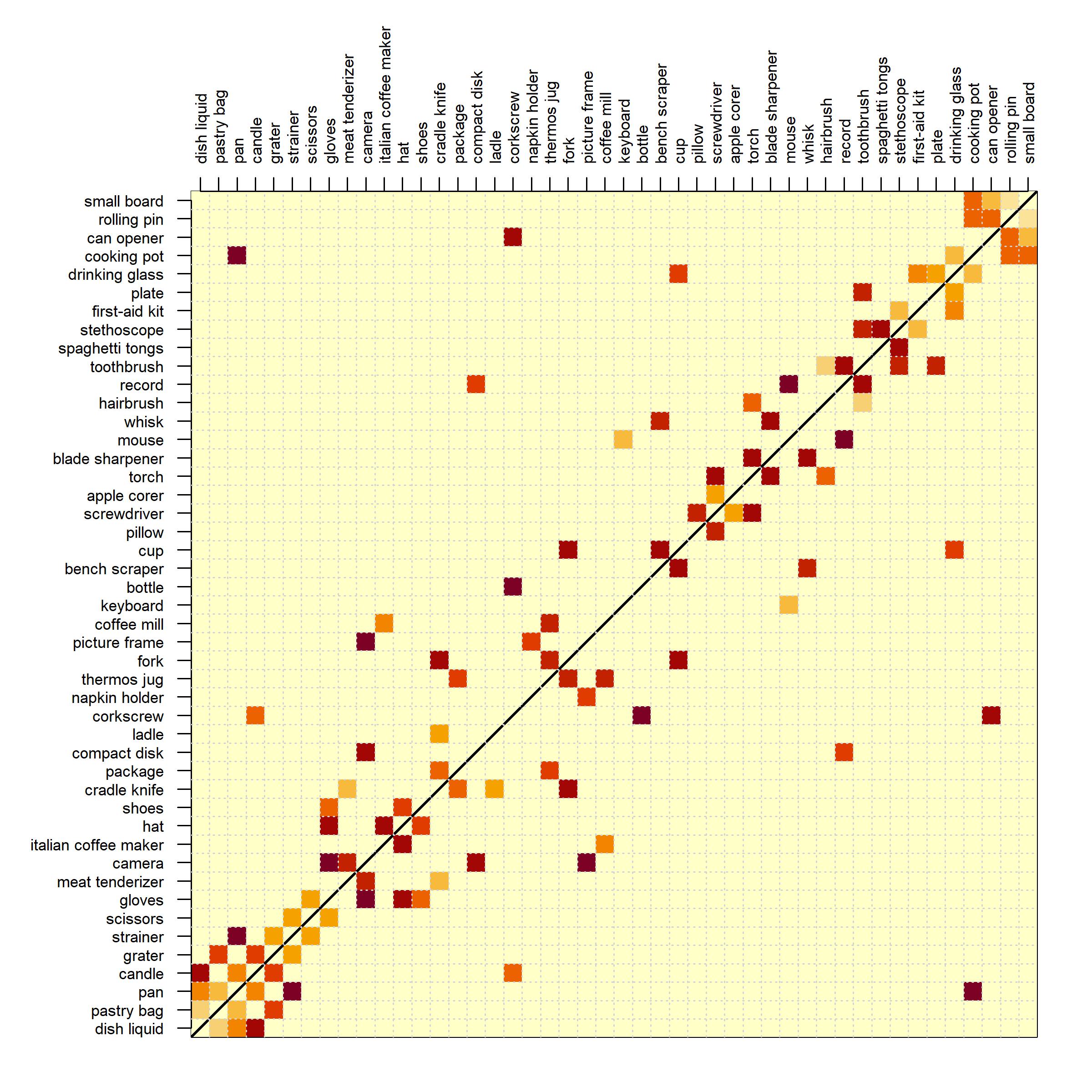

Supplement: Supplementary file 7 [file Image_5.JPEG]
